# Embryonic Heat Conditioning Induces TET-Dependent Cross-Tolerance to Hypothalamic Inflammation Later in Life

**DOI:** 10.3389/fgene.2020.00767

**Published:** 2020-08-05

**Authors:** Tali Rosenberg, Tatiana Kisliouk, Tomer Cramer, Dmitry Shinder, Shelly Druyan, Noam Meiri

**Affiliations:** ^1^Agricultural Research Organization, Volcani Center, Institute of Animal Science, Rishon LeZion, Israel; ^2^Department of Animal Science, The Robert H. Smith Faculty of Agriculture, Food and Environment, The Hebrew University of Jerusalem, Rehovot, Israel

**Keywords:** embryonic heat conditioning, epigenetics, TET, hypothalamus, chicks

## Abstract

Early life encounters with stress can lead to long-lasting beneficial alterations in the response to various stressors, known as cross-tolerance. Embryonic heat conditioning (EHC) of chicks was previously shown to mediate resilience to heat stress later in life. Here we demonstrate that EHC can induce cross-tolerance with the immune system, attenuating hypothalamic inflammation. Inflammation in EHC chicks was manifested, following lipopolysaccharide (LPS) challenge on day 10 post-hatch, by reduced febrile response and reduced expression of *LITAF* and *NF*κ*B* compared to controls, as well as nuclear localization and activation of NFκB in the hypothalamus. Since the cross-tolerance effect was long-lasting, we assumed that epigenetic mechanisms are involved. We focused on the role of ten-eleven translocation (TET) family enzymes, which are the mediators of active CpG demethylation. Here, TET transcription during early life stress was found to be necessary for stress resilience later in life. The expression of the TET family enzymes in the midbrain during conditioning increased in parallel to an elevation in concentration of their cofactor α-ketoglutarate. *In-ovo* inhibition of TET activity during EHC, by the α-ketoglutarate inhibitor bis-2-(5-phenylacetamido-1,3,4-thiadiazol-2-yl) ethyl sulfide (BPTES), resulted in reduced total and locus specific CpG demethylation in 10-day-old chicks and reversed both thermal and inflammatory resilience. In addition, EHC attenuated the elevation in expression of the stress markers *HSP70*, *CRHR1*, and *CRHR2*, during heat challenge on day 10 post-hatch. This reduction in expression was reversed by BPTES. Similarly, the EHC-dependent reduction of inflammatory gene expression during LPS challenge was eliminated in BPTES-treated chicks. Thus, TET family enzymes and CpG demethylation are essential for the embryonic induction of stress cross-tolerance in the hypothalamus.

## Introduction

All stressful exposures have an impact on the brain, regardless of whether they occur during the pre- or postnatal period, infancy or adulthood (Lupien et al., 2009). Emerging scientific consensus argues that the origins of adult disease, psychopathologies included, are often found among developmental and biological disruptions occurring during development and the early years of life. Either biological embedding of adversities during sensitive developmental periods, or accumulated damage gathered over time, can severely influence the formation of such diseases during adulthood (Shonkoff et al., 2009; Taylor, 2010; Labonté et al., 2012). These embryonic or early life environmental influences can lead to different stress responses, including vulnerability or resilience. Since these environmental effects are long-lasting, epigenetic regulatory mechanisms are probably involved and are key factors in the establishment and tuning of adult stress responses (Klengel and Binder, 2015; Turecki and Meaney, 2016; Horowitz, 2017; Cramer et al., 2018). The acquired stress response can have a broader impact than merely an adjusted response to the encountered stressor; it can also have a cross effect with other stress occurrences.

Cross-tolerance describes resilience to one stressor acquired by conditioning to a different stressor. For example, heat acclimation has been shown to improve cardiovascular function as well as protect against ischemia (Horowitz et al., 2015; Pollak et al., 2017). Here, we were interested in the cross-tolerance effect of embryonic heat conditioning (EHC) of chicks. The chick is an optimal model for studying manipulations during the embryonic period, because embryonic manipulations can be performed without affecting the maternal environment, and the chick is completely independent of maternal guidance after hatch (Haller et al., 2015; Goodfellow et al., 2016; Duman et al., 2018). EHC performed during the time of hypothalamic–pituitary–adrenal (HPA) axis development has been shown to improve thermal tolerance later in life (Piestun et al., 2009). Although the HPA axis has been shown to be involved in the development of stress, it has also been implicated in central nervous system (CNS) inflammation (Hueston and Deak, 2014). Here, EHC was used to induce cross-tolerance with hypothalamic inflammation later in life.

The hypothalamus is a major contributor to the development of stress responses (Smith and Vale, 2006), but it is also a key regulator of energy and thermal homeostasis (Morrison, 2016). Various studies have demonstrated that early life stress is a contributing factor to adult inflammation (Pace et al., 2006; Miller et al., 2009; Carpenter et al., 2010; Gouin et al., 2012; Fagundes et al., 2013). Hypothalamic inflammation is key during the onset of obesity (Roesslein et al., 2015; André et al., 2017; Jais and Brüning, 2017), and aggravates the neurological damage incurred from stroke, heat stroke, or traumatic brain injury (Arvin et al., 1996; Emsley et al., 2008; Macrez et al., 2011; Chao et al., 2015). In this work, we studied the effect of EHC on hallmarks of CNS inflammation in the hypothalamus.

CNS inflammation involves activation of TLR4/MyD88 nuclear factor kappa B (NFκB)-dependent transcription of inflammatory cytokines, such as tumor necrosis factor alpha (TNFα), interleukin (IL) 1β and IL6, and an increase in nitric oxide (NO) production in the resident microglia (Wang et al., 2009; Tang and Le, 2016). TLR4 dependent NO production by resident microglia is triggered by nitric oxide synthase 2 (NOS2) which is expressed in neurons (Béchade et al., 2014). Moreover this inflammatory activation of NO leads to detrimental protein radical formation (Kumar et al., 2014). CNS inflammation is also characterized by blood brain barrier disruption derived by astrocyte production of vascular Endothelial Growth Factor A (VEGFA) (Argaw et al., 2012). Alternative, anti-inflammatory activation of microglia, characterized by the increased expression of cytokines IL4 and IL10, as well as mannose receptor C-type 1 (MRC1), and increased arginase activity, has been shown to be necessary for anti-inflammatory effects, as well as parasite clearance, allergic responses and angiogenesis (Martinez and Gordon, 2014). To study the effect of EHC on hypothalamic inflammation, we injected chicks with lipopolysaccharide (LPS) and measured the gene expression of the proinflammatory LPS-induced TNF factor (*LITAF*) and *NF*κ*B*, as well as the anti-inflammatory *IL10* and *MRC1*. Previous study in laying hens, demonstrated that early life (5 days of age) heat stress had no effect on LPS reaction, later in life (Star et al., 2009), however very little is known regarding LPS reaction in the EHC model.

We focused on the epigenetic regulation during EHC that is implicated in creating cross-tolerance with reduced hypothalamic inflammation. Epigenetics is the study of factors, mostly environmental, that affect gene expression without changing the genetic sequence. There are different layers of epigenetic regulation, including DNA methylation inhibiting the association of enhancing or inhibitory elements, histone-tail modifications such as acetylation or methylation that change the gene’s accessibility to trans elements, and microRNA regulation of transcription (Meaney and Szyf, 2005; Goldberg et al., 2007). CpG methylation is performed by DNA methyl transferases (DNMT’s). This family of enzymes include DNMT1 that preserves DNA methylation during development (Hoffmann et al., 2015), DNMT3A and DNMT3B that perform *de novo* DNA methylation (Hoffmann et al., 2015). Recently, hydroxymethylation, leading to active CpG demethylation by the ten-eleven translocation (TET) family enzymes, was discovered (Kohli and Zhang, 2013). TET proteins have three main isoforms (TET1-3), all sharing a C-terminal catalytic domain with the ability to localize to the nucleus and perform 5mC oxidation. However, only full length TET1 and TET3 also contain an N-terminal CXXC, DNA binding domain. TET2 lost its CXXC binding domain due to genomic inversion during evolution, forming the adjacent gene IDAX, which enables TET2 DNA binding, but also mediates TET2 degradation. Moreover, TET proteins also display various catalytic independent regulation of gene transcription, mostly by association with histone modifiers (Wu and Zhang, 2017; Melamed et al., 2018). TET1 expression is high in embryonic stem cells (ESC) and primordial germ cells (PGC), moreover, upon induction of pluripotency, both TET1 and TET2 expression is increased (Branco et al., 2012). TET2 mutations are abundant in hematopoietic malignancies, and cardiovascular diseases (Ferrone et al., 2020), as well as in increased inflammatory macrophage activation in TET2 knockouts (Fuster et al., 2017). TET3 expression peaks in oocytes and zygotes (Branco et al., 2012), and also in neurons (Wu and Zhang, 2017). Interestingly, DNMT3A, a ***de novo***, DNA methyltransferase, inhibits TET2 and TET3 in hematopoietic malignancies (Gong et al., 2016). Epigenetic processes are influenced by the metabolic environment (Ruben et al., 2015) and different metabolites, such as acetyl-CoA, **α** -ketoglutarate, NAD **+** and S-adenosylmethionine serve as regulators of epigenetic enzymes such as histone deacetylase, DNA methyltransferase and TET family enzymes (Baardman et al., 2015).

TET family enzymes have been reported to regulate different types of inflammation (Yang et al., 2016; Vento-Tormo et al., 2017; Hassan et al., 2018), as well as contribute to macrophage polarization and activation (Wallner et al., 2016). Although changes in DNA methylation during CNS inflammation have been previously reported (Ligthart et al., 2016), little is known about the role of TET family enzymes during CNS inflammation. Recently, the role of TET in the hypothalamus was explored during postnatal development in mice. Although *TET* expression was downregulated from birth to postnatal day 25, DNA hydroxymethylation increased (Cisternas et al., 2020), indicating a possible role during embryonic development of the hypothalamus. TET enzymes were also necessary to promote heat-stress resilience in chicks (Cramer et al., 2018, 2019). Here we demonstrate the role of TET family enzymes in the hypothalamus, during EHC, in the establishment of cross-tolerance to hypothalamic inflammation later in life.

## Materials and Methods

### Experimental Model and Subject Details

EHC was performed as previously described (Piestun et al., 2015). Briefly, we used 420 fertile Cobb strain broiler (*Gallus domesticus*) eggs from one breeder flock of hens. All eggs were obtained from Brown hatcheries (Hod Hasharon, Israel). The eggs were arbitrarily divided into two incubation treatment groups without application of specific randomization methods: control – eggs were incubated at 37.8°C and 56% relative humidity throughout the 21 days of the incubation period; EHC – eggs were incubated at 39.5°C and 65% relative humidity for 12 h/day from embryonic day (ED) 7 to ED 16. The eggs were incubated in two type 65Hs automatic incubators (Masalles, Barcelona, Spain).

Hatched chicks were arbitrarily divided into pens, eight chicks per pen, in climate-controlled rooms at 32°C under a 22/2 h cycle of artificial illumination with *ad libitum* access to food and water. On day 7 post-hatch, the temperature was changed to 30°C and chicks were further divided into five chicks per pen. On day 10 post-hatch, the chicks were subjected to either heat or LPS challenge. A previous experiment did not show any differences related to the sexes, so we did not distinguish between sexes in this work.

Heat challenge was performed as previously described (Kisliouk et al., 2017). Briefly, on day 10 post-hatch, both control and EHC experimental chick groups were thermally challenged by exposure to 36°C for 24 h. The chicks’ baseline body temperature was measured and they were sacrificed by decapitation 0, 2, 6, and 24 h into the heat challenge.

For the LPS challenge, 0.3 μg LPS (Sigma Aldrich, Rehovot, Israel) originated from *Salmonella* and its vehicle solution (0.9% w/v NaCl, designated as saline), were administered by intracerebroventricular (ICV) injection into both control and EHC experimental chick groups. Since the brain has no pain receptors, and we wanted to minimize animal stress and suffering, no anesthetics were used for the ICV injections. The chicks’ body temperature was measured and they were sacrificed by decapitation 0 and 6 h into the LPS challenge.

All challenges were initiated at 07:00 h and continued for 24 or 6 h. Temperature measurements during the challenges were conducted blindly. To minimize stress and suffering, animals were killed by fast decapitation.

### Embryonic Inhibition of Glutaminase

Chorioallantoic membrane of ED 12 embryos was injected with the glutaminase inhibitor bis-2-(5-phenylacetamido-1,3,4-thiadiazol-2-yl) ethyl sulfide (BPTES) (Tocris, Bristol, United Kingdom) dissolved in DMSO and further diluted in 0.9% (w/v) NaCl to a final concentration of 0.01% (v/v) DMSO. Each embryo received 12.5 mg/kg BPTES–0.01% DMSO dissolved in 0.9% NaCl (designated BPTES) or 0.01% DMSO dissolved in 0.9% NaCl (designated saline). Immediately following injection, eggs were sealed with hot glue and returned to their incubators.

### Tissue Collection

For total RNA and DNA isolation, the brain area matching the anterior hypothalamus was dissected and immersed in RNALater pH 5.2 (5.3 M ammonium sulfate, 25 mM sodium citrate, 20 mM EDTA). For western blots, α-ketoglutarate quantification, TET activity assay and global 5mC and 5hmC, isolated tissues were immediately frozen in liquid nitrogen and stored at −80°C.

### Total RNA Isolation and Quantitative PCR

Total RNA was isolated using TRI Reagent (Molecular Research Center, Cincinnati, OH, United States) according to the manufacturer’s instructions. Hypothalamic RNA (0.5 μg) was reverse-transcribed to single-stranded cDNA by Super Script II Reverse Transcriptase and oligo (dT) plus random primers (Thermo Fisher Scientific, Waltham, MA, United States). Quantitative PCR was performed with 10 ng cDNA in a StepOnePlus Real Time PCR System (Applied Biosystems) with PerfeCta SYBR Green FastMix, ROX (Quanta BioSciences, Gaithersburg, MD, United States). Dissociation curves were analyzed following each real-time PCR to confirm the presence of only one product and the absence of primer dimer formation. The threshold cycle number (Ct) for each tested gene (X) was used to quantify the relative abundance of that gene using the formula [2^–(Ct gene X^ ^–Ct standard)^]. Hydroxymethylbilane Synthase (HMBS) was used as the standard for mRNA expression. The primers used for real-time PCR were as follows (5′→3′):

**Table:** 

**Gene name**	**Primer FW**	**Primer REV**
*HMBS* (Cramer et al., 2018)	CGTTTGGAGGGTGGCTGTAG	TGTCAAGTACAACTGGCCATCTTT
*HSP70* (Kisliouk et al., 2017)	TGGGTGTCTTCCAGCATGG	GATGAGGCGCTCTGTATCGG
*BDNF* (Kisliouk and Meiri, 2009)	GCTTGGCTTACCCAGGTCTTC	TCAAAAGTGTCCGCCAGTG
*GILZ*	CATGGAGGTGGCTGTCTATCAG	GGCACTGTTGTCCAGCTTCA
*EIF2B5* (Kisliouk et al., 2009)	GAAATCCAAGTGGTGCCG	GCATCAACATCTCGCAGCA
*CRHR1* (Kadhim et al., 2019)	CCCTGCCCCGAGTATT TCTA	CTT GCTCCTCTTCTCCTCACTG
*CRHR2* (Kadhim et al., 2019)	GCAGTCTTTTCAGGGTTTCTTTG	CGGTGCCATCTTTTCCTGG
*LITAF*	CCAGATGGGAAGGGAATGAA	CTACGGGTTGCTGCACATACA
*NFkB* (Tan et al., 2014)	GTGTGAAGAAACGGGAACTG	GGCACGGTTGTCATAGATGG
*NOS2*	GATTGGGTGTGGATTGTACC	TGCATCCACCTGGTAGTAAA
*VEGFA*	ACAAGAAAATCACTGTGAGCC	ACGTGAGTCTGTGAATTTGC
*IL10*	TTAACCCACTGCCTCTCACC	CCTTCTCGAACGTCTCCTTG
*MRC1*	TGGGTCTCAGATCACCAGCTT	GAGTGACCGGAACCCAGTCTT
*TET1* (Cramer et al., 2019)	AATTCCGCACCCACAGTTAC	CCAAATCCCCATCTCCTTTT
*TET2* (Cramer et al., 2019)	GCAAATTATTGAAAAAGATGAAGG	CGGCTCCTAGGTGGGTATAG
*TET3* (Cramer et al., 2019)	CCACTGCCAGAATGCTGTCA	GGTACAGTGTGTCGCCCAGAGT
*DNMT1* (Cramer et al., 2019)	CCCGACC CCTATCGTGTG	AACTTCCAGATGCGCAG TTTG
*DNMT3A* (Cramer et al., 2019)	AGTACCTCTCCATCTCCGTGCA	CACTCGCTCCAACTCCATCAA
*DNMT3B* (Cramer et al., 2019)	CCTGGAGTGTAACCCC GTTATG	CAAAGATCCTGTTCATCCCTGG

### Preparation of Cytosolic and Nuclear Fractions and Western Blotting

Frozen anterior hypothalamus was lysed in PBS containing protease and phosphatase inhibitors (Sigma). Lysates were centrifuged at 1000 *g* for 8 min at 4°C. The supernatant was added to Laemmli sample buffer and marked as the cytosolic fraction. The pellet was further lysed in nuclear extraction buffer (20 mM Tris pH 7.8, 125 mM NaCl, 5 mM MgCl_2_, 0.2 mM EDTA, 12% glycerol, 0.1% NP-40 and protease and phosphatase inhibitors), sonicated and centrifuged at 1000 *g* for 8 min at 4°C. The supernatant was added to Laemmli sample buffer and marked as the nuclear fraction. Cytosolic and nuclear fractions were loaded onto a polyacrylamide gel for electrophoresis, then transferred to a nitrocellulose membrane and blocked with 3% skim milk (Sigma) for 1 h at room temperature. Membranes were incubated overnight with primary antibodies against NFκB (1:500, Abcam, Cambridge, United Kingdom, PRID: AB_443394), IKKα/β (1:500; Biorbit, Cambridge, United Kingdom, PRID: AB_2783519), p-Ser 176/177–IKKα + β (1:500, Life Span Biotechnology, Mandideep, Bhopal, India, PRID: AB_2783518), actin (1:6000; Cell Signaling Technology, Beverly, MA, United States, PRID: AB_330288) and H3 (1:2000, Cell Signaling Technology, PRID: AB_1904005), followed by three washes and incubation with secondary anti-rabbit IgG horseradish peroxidase-conjugated antibody (1:5000; Amersham Biosciences, Little Chalfont, United Kingdom, PRID: AB_772206) for 1 h at room temperature; after an additional three washes, membranes were reacted with SuperSignal Chemiluminescent substrates according to the manufacturer’s instructions (Pierce Biotechnology, Rockford, IL, United States). Blots were imaged by the G:BOX chemi XRQ gel-imaging system (Syngene – Synoptics Ltd., Cambridge, United Kingdom), and densitometric analysis was performed using Quantity One 1-D analysis software (Bio-Rad, Hercules, CA, United States).

### α-Ketoglutarate Quantification

All of the samples were analyzed for α-ketoglutarate quantification in the same experiment, using Alpha Ketoglutarate (alpha KG) Assay Kit according to the manufacturer’s protocols (Abcam).

### TET Activity

TET activity was measured in the midbrain of chick embryos on ED 14 using the Epigenase 5mC-Hydroxylase TET Activity/Inhibition Assay Kit according to the manufacturer’s protocols (EpiGentek, Farmingdale, NY, United States).

### DNA Methylation and Hydroxymethylation Analyses

Total DNA methylation (%5mC) and hydroxymethylation (%5hmC) were assessed using “MethylFlash” global DNA methylation ELISA kit and “MethylFlash” global DNA hydroxymethylation ELISA kit, accordingly (EpiGentek).

Sequence specific DNA methylation (%5mC) and hydroxymethylation (%5hmC) was assessed using the “EpiMark 5-hmC and 5-mC Analysis kit” (NEB, Ipswich, MA) according to the manufacturer’s protocol. Briefly, DNA from day 10 post-hatch anterior hypothalamus, was incubated with T4 β-glucosyltransferase (T4-BGT, which adds a glucose mark on 5hmC, creating 5ghmC), 37°C, overnight. T4-BGT^+^ and T4-BGT^–^ samples were than incubated with *Msp*I (cleaves DNA at CCGG, unless the middle CG is 5ghmC) or *Hpa*II (cleaves DNA at CCGG unless the middle CG is modified to 5mC, 5hmC, or 5ghmC) restriction enzymes, 37°C, 12 h. The restriction products are later incubated with proteinase K, 40°C, 30 min, followed by 10 min of 95°C for proteinase K inactivation. DNA samples were used in qPCR to determine %5hmC and %5mC according to the expression of specific targets.

$$\%5hmC=\frac{\left(t_{5}-t_{1}\times \left(t_{6}:t_{3}\right)\right)}{t_{6}}\times 100,\%5mC=\frac{\left(t_{1}\times \left(t_{6}:t_{3}\right)\right)}{t_{6}}\times 100$$

t1 = expression of T4-BGT^+^, Msp^+^; t3 = expression of T4-BGT^+^, uncut; t4 = expression of T4-BGT^–^, Msp^+^; t5 = expression of T4-BGT^–^, Hpa^+^; t6 = expression of T4-BGT^–^, uncut.

For qPCR we designed primers amplifying regions containing CCGG, which presented increased probability for binding transcription factors via the TFBIND algorithm^1^.

**Table:** 

**Gene name**	**Primer FW**	**Primer REV**
*LITAF*	AGCGAGTGCCGGTGG	CGACCCGGAGCAGTGAG
*NFkB*	GAAGCGCCGCCGGTT	CGACCTTCCTCCGGGCA
*GILZ*	ACAAGGTCACCCGGCT	ATAGGGACGCCGGGG

### Quantification and Statistical Analysis

Statistical analysis was performed using GraphPad Prism 6 software (GraphPad Software, San Diego, CA, United States). All data were examined for normality by goodness of fit test and by Bartlett test for variance equality. As the distribution was normal, the parameters were not transformed. There was no sample size calculation. Nevertheless, the sample size was determined on the basis of previous studies (Kisliouk et al., 2017; Cramer et al., 2018). Sample number (n) included the number of individual chicks in each treatment group (indicated in Figure Legends). Means of two groups were compared by two-tailed unpaired *t*-test. Comparisons of LPS and conditioning effects were analyzed by two-way ANOVA followed by Sidak’s or LSD multiple-comparison tests. Figure data are presented as mean ± SEM, with exact n and *P*-values reported in the legends. The “Results” section provides the *F* and *t*-test expressions.

## Results

### Cross-Tolerance: EHC Attenuates the Inflammatory Response in the Hypothalamus

**FIGURE 1:**
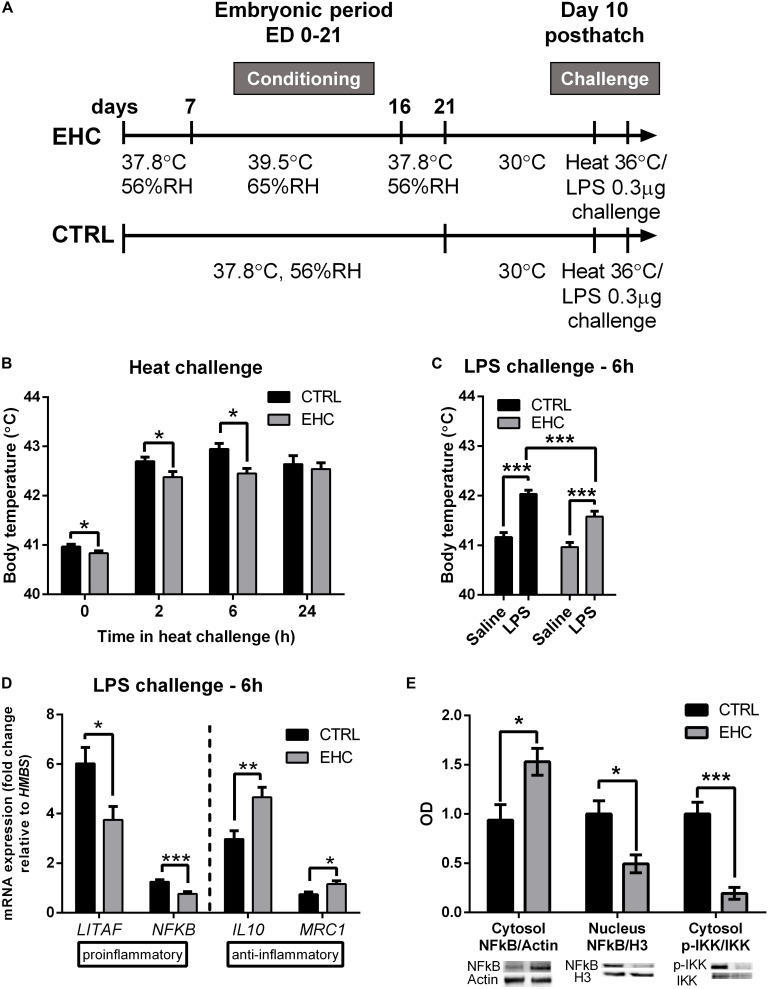
Cross-tolerance: embryonic heat conditioning (EHC) attenuates the inflammatory response in the hypothalamus. **(A)** Schematic representation of the experiment. EHC on embryonic days (ED) 7–16, followed by heat/LPS challenge on day 10 post-hatch. **(B)** Body temperature measured 10 days post-hatch and at different times (0, 2, 6, 24 h) into the heat challenge (36°C). t_0_ (n_*CTRL*_ = 23, n_*EHC*_ = 27, *P = 0.047); 2 h (n_*CTRL*_ = 26, n_*EHC*_ = 27, *P = 0.026); 6 h (n_*CTRL*_ = 25, n_*EHC*_ = 27, **P = 0.002); 24 h (n_*CTRL*_ = 17, n_*EHC*_ = 17, *P* = 0.64). **(C)** Chicks (10 days old) were ICV-injected with LPS (0.3 μg) or saline vehicle and their body temperature was measured 6 h after the injection. CTRL (n_*saline*_ = 30, n_*LPS*_ = 29, ***P < 0.0001); EHC (n_*saline*_ = 27, n_*LPS*_ = 26, ***P < 0.0001); LPS (n_*CTRL*_ = 29, n_*EHC*_ = 26, **P = 0.005). **(D)** Hypothalamic mRNA expression 6 h after LPS or saline injection; fold change of *LITAF*, *NF*κ*B*, *IL10*, and *MRC1* was normalized to *HMBS*, and reported relative to their respective saline controls. *LITAF* (n_*CTRL*_ = 25, n_*EHC*_ = 20, *P = 0.012); *NF*κ*B* (n_*CTRL*_ = 25, n_*EHC*_ = 20, ***P = 0.0003); *IL10* (n_*CTRL*_ = 22, n_*EHC*_ = 19, **P = 0.002); *MRC1* (n_*CTRL*_ = 6, n_*EHC*_ = 6, *P = 0.03). **(E)** Western blotting of cytosolic and nuclear fractions against NFκB, and cytosolic IKKα/β and p-IKKα/β, 6 h into LPS challenge. Cytosolic fraction proteins were normalized to actin and nuclear fraction proteins were normalized to histone 3 (H3). Bottom panel shows the representative blots; top panel presents western quantification. Nuclear localization of NFκB/H3 (n_*CTRL*_ = 4, n_*EHC*_ = 4, *P = 0.02), cytosolic localization in the EHC group (*P = 0.03), cytosolic IKKα/β phosphorylation (***P = 0.0009). Data are presented as mean ± SEM, and significant effects between groups are indicated as *0.05 < *P* < 0.01, **0.01 < *P* < 0.001, ***P < 0.001 using ANOVA test with LSD for multiple comparisons. CTRL, Control; RH, relative humidity.

Chicks were heat-conditioned between ED 7 and 16. After hatch, chicks were raised in their optimal environmental temperature of 30°C. Ten days after hatch, both EHC and control (non-conditioned) chicks were subjected to heat (36°C) or LPS (0.3 μg) challenge (Figure 1A). As a result of EHC, the body temperature of the EHC group on day 10 post-hatch was significantly lower than that of the control group (*t*_47_ = 2.03, *P* = 0.047). The EHC group’s body temperature remained significantly lower than that of the control group 2 and 6 h into the heat challenge (*t*_52_ = 2.28, *P* = 0.026; *t*_51_ = 3.27, *P* = 0.002, respectively) (Figure 1B). LPS challenge was performed on day 10 post-hatch by ICV injection of either LPS or saline. As expected, LPS induced a febrile response in both groups (LPS effect *F*_1_, _108_ = 64.16, *P* < 0.0001), but the body temperature of the EHC–LPS group was significantly lower than that of the control–LPS group 6 h into the challenge (*P* = 0.005; Figure 1C), indicating that the embryonic conditioning attenuated the febrile response during the LPS challenge (conditioning effect *F*_1, 108_ = 12.59, *P* = 0.0006).

To determine whether the reduced febrile response to LPS is accompanied by reduced inflammation, we analyzed the expression patterns of both pro- and anti-inflammatory genes from the hypothalamic tissue of chicks sacrificed 6 h into the LPS challenge (Figure 1D). Following LPS challenge, the expression of the proinflammatory genes *LITAF* and *NF*κ*B* was significantly reduced in the EHC group compared to controls (*LITAF* by 38%, *t*_43_ = 2.63, *P* = 0.01; *NF*κ*B* by 39%, *t*_43_ = 3.91, *P* = 0.0003). Furthermore, there was a significant increase in the expression of the anti-inflammatory genes *IL10* and *MRC1* (*IL10* by 56%, *t*_39_ = 3.28, *P* = 0.002; *MRC1* by 57%, *t*_30_ = 2.57, *P* = 0.03; Figure 1D). Translocation of NFκB protein to the nucleus was also measured 6 h after LPS injection. Its cytosolic levels were increased in the EHC group (*t*_6_ = 2.83, *P* = 0.03), and its nuclear levels were significantly reduced (*t*_6_ = 3.11, *P* = 0.02). Furthermore, IKKα/β, which phosphorylates the regulatory subunit of NFκB to enable its nuclear translocation, and phosphorylated on its own Ser176/177, indicating IKKα/β activity (Gilmore, 2006), was less evident in the EHC group (*t*_6_ = 6.07, *P* = 0.0009; Figure 1E).

### TET Family Enzymes Present Increased Expression During EHC

**FIGURE 2:**
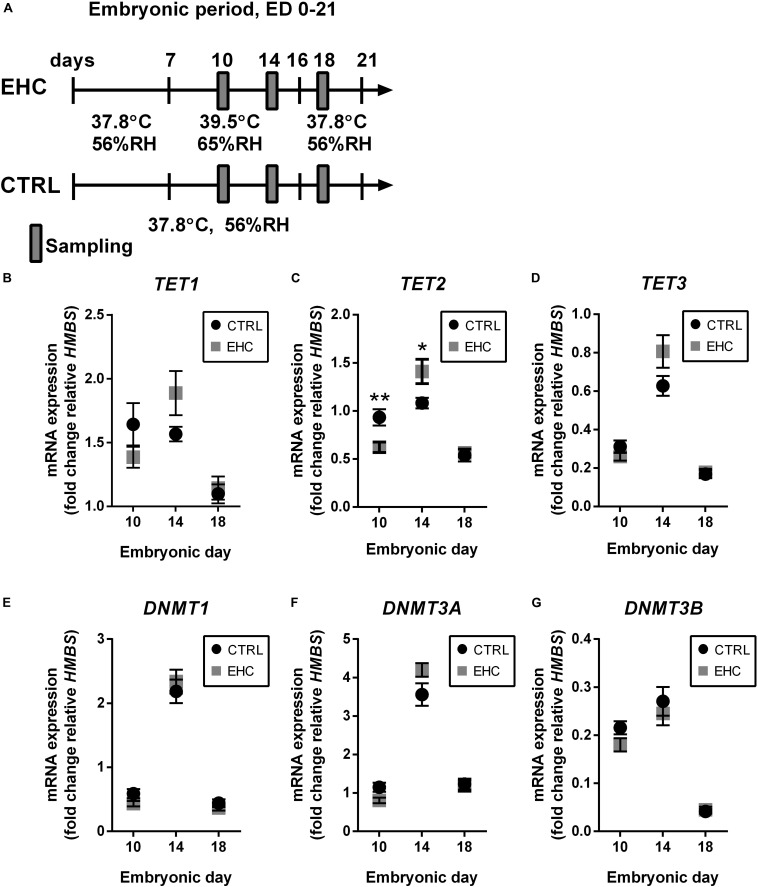
TET family enzymes present increased expression during EHC. **(A)** Experimental scheme. Midbrain samples were taken on embryonic days (ED) 10 and 14 (during conditioning), and on ED 18 (after conditioning). **(B)** mRNA expression of *TET1* did not differ between control (CTRL) and EHC groups on ED 10 (n_*CTRL*_ = 10, n_*EHC*_ = 10, *P* = 0.18), 14 (n_*CTRL*_ = 9, n_*EHC*_ = 8, *P* = 0.08) or 18 (n_*CTRL*_ = 10, n_*EHC*_ = 10, *P* = 0.7). **(C)** mRNA expression of *TET2* was significantly lower in the EHC vs. CTRL group on ED 10 (n_*CTRL*_ = 10, n_*EHC*_ = 10, **P = 0.006), but significantly higher on ED 14 (n_*CTRL*_ = 9, n_*EHC*_ = 8, *P = 0.03), and did not differ on ED 18 (n_*CTRL*_ = 10, n_*EHC*_ = 10, *P* = 0.73). **(D)** mRNA expression of *TET3* did not differ between CTRL and EHC groups on ED 10 (n_*CTRL*_ = 10, n_*EHC*_ = 10, *P* = 0.2), 14 (n_*CTRL*_ = 9, n_*EHC*_ = 8, *P* = 0.09) or 18 (n_*CTRL*_ = 10, n_*EHC*_ = 10, *P* = 0.74). **(E)** mRNA expression of *DNMT1* did not differ between CTRL and EHC groups on ED 10 (n_*CTRL*_ = 10, n_*EHC*_ = 10, *P* = 0.07), 14 (n_*CTRL*_ = 9, n_*EHC*_ = 8, *P* = 0.57) or 18 (n_*CTRL*_ = 10, n_*EHC*_ = 10, *P* = 0.32). **(F)** mRNA expression of *DNMT3A* was significantly lower in the EHC vs. CTRL group on ED 10 (n_*CTRL*_ = 10, n_*EHC*_ = 10, *P = 0.02), but did not differ on ED 14 (n_*CTRL*_ = 9, n_*EHC*_ = 8, *P* = 0.09), and did not differ on ED 18 (n_*CTRL*_ = 10, n_*EHC*_ = 10, *P* = 0.81). **(G)** mRNA expression of *DNMT3B* did not differ between CTRL and EHC groups on ED 10 (n_*CTRL*_ = 10, n_*EHC*_ = 10, *P* = 0.08), 14 (n_*CTRL*_ = 9, n_*EHC*_ = 8, *P* = 0.53) or 18 (n_*CTRL*_ = 10, n_*EHC*_ = 10, *P* = 0.71). Data are presented as mean ± SEM, and significant effects between groups are indicated by *0.05 < *P* < 0.01, **0.01 < *P* < 0.001. RH, relative humidity.

Since we showed that EHC has a long-term effect on the expression of inflammatory genes, we assumed that epigenetic mechanisms are involved. Therefore, we measured mRNA expression of both the *TET* and the *DNMT* family enzymes in the midbrain during EHC on ED 10 and 14, and after the end of conditioning on ED 18 (Figure 2A). *TET1* expression did not differ between EHC and control groups on ED 10 (*t*_18_ = 1.4, *P* = 0.18), 14 (*t*_15_ = 1.9, *P* = 0.08), or 18 (*t*_18_ = 0.39, *P* = 0.7). However, during conditioning, average *TET1* expression in the EHC group increased from ED 10 to ED 14 (by 36%), while control values remained constant. Moreover, *TET1* expression on ED 18, after conditioning, was lower in both control (by 34% compared to ED 10) and EHC (by 17% compared to ED 10; Figure 2B) groups. On ED 10, *TET2* expression in the EHC group was significantly lower than in the control group (*t*_18_ = 3.1, *P* = 0.006). However, on ED 14, *TET2* expression was higher in the EHC group compared to the control group (*t*_15_ = 2.5, *P* = 0.03). There was no difference in *TET2* expression after the conditioning, on ED 18, between EHC and control groups (*t*_18_ = 0.34, *P* = 0.74). Similar to *TET1* expression, *TET2* expression in the EHC group presented an increase between ED 10 and ED 14 (by 127%), while the control, non-conditioned group displayed similar expression between ED 10 and ED 14. After the conditioning period on ED 18, *TET2* expression returned to its level on ED 10 in the EHC group, and was lower for the control group (43%; Figure 2C). *TET3* expression did not differ between EHC and control groups on any of the experimental days. During the conditioning period, both groups presented increased *TET3* expression between ED 10 and ED 14 (EHC by 200% and control by 100%), and *TET3* expression on ED 18, after the conditioning period, was reduced in both control (40%) and EHC (48%) groups relative to its level on ED 10 (Figure 2D). Overall, TET enzymes presented dynamic expression during the conditioning period, which may indicate their role during thermal conditioning and hypothalamic development. *DNMTs* expression did not differ between CTRL (non-conditioned) and EHC groups on all days of measurement except for DNMT3A on ED 10. *DNMT1* expression increased between ED 10 and ED 14, and returned to baseline expression on ED 18 (ED 10: *t*_18_ = 1.90; *P* = 0.07; ED 14: *t*_15_ = 0.58; *P* = 0.57; ED 18: *t*_18_ = 1.02, *P* = 0.32; Figure 2E). *DNMT3A* expression was significantly lower in the EHC group on ED 10 (*t*_18_ = 2.49; *P* = 0.02) and its expression on ED 14 tended to increase compared to CTRL group (*t*_15_ = 1.81; *P* = 0.09). There was no difference in *DNMT3A* expression during ED 18 (*t*_18_ = 0.24, *P* = 0.81; Figure 2F). *DNMT3B* expression did not differ between CTRL and EHC groups on all days of measurement (ED 10: *t*_18_ = 1.86; *P* = 0.08; ED 14: *t*_15_ = 0.64; *P* = 0.53; ED 18: *t*_18_ = 0.38, *P* = 0.71; Figure 2G).

### TET Enzyme Cofactor α-Ketoglutarate Concentration in the Midbrain Is Increased and Necessary for TET Activity During EHC

**FIGURE 3:**
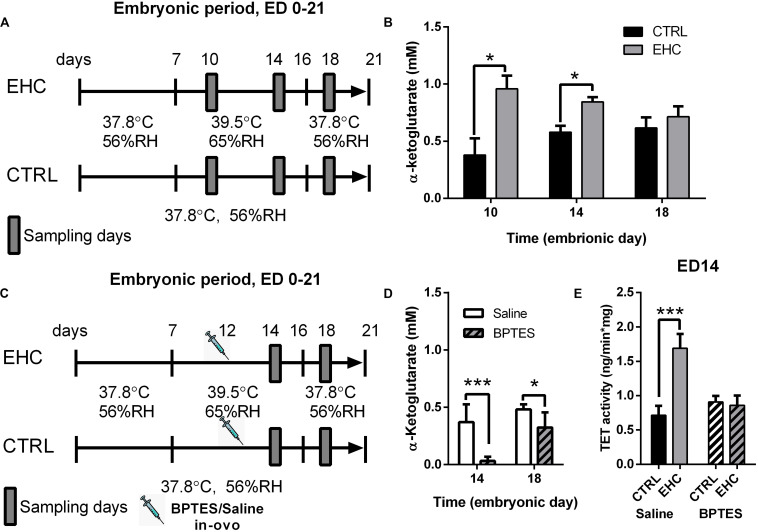
TET enzyme cofactor α-ketoglutarate concentration in the midbrain is increased and necessary for TET activity, during EHC. **(A)** Experimental scheme. Midbrain samples were taken on embryonic days (ED) 10 and 14 (during conditioning), and on ED 18 (after conditioning). **(B)** α-Ketoglutarate concentration (mM) in the midbrain of chick embryos on ED 10, 14, and 18. ED 10 (n_*CTRL*_ = 4, n_*EHC*_ = 4, *P = 0.02); ED 14 (n_*CTRL*_ = 8, n_*EHC*_ = 8, **P = 0.002); ED 18 (n_*CTRL*_ = 7, n_*EHC*_ = 7, *P* = 0.46). **(C)** Experimental scheme; 12.5 mg/kg BPTES or saline vehicle was injected on ED 12. **(D)** α-Ketoglutarate concentration (mM) in the midbrain of chicks injected with BPTES (*n* = 8) or saline (*n* = 8). ED 14 (***P = 0.0002); ED 18 (*P = 0.02). **(E)** TET activity in the midbrain on ED 14. Saline-injected group (n_*EHC*_ = 5, n_*CTRL*_ = 4, ***P < 0.0001); BPTES-injected group (n_*EHC*_ = 5, n_*CTRL*_ = 5, *P* = 0.99). Data are presented as mean ± SEM, and significant effects between groups are indicated by *0.01 < *P* < 0.05, **0.001 < *P* < 0.01, ***P < 0.001. CTRL, control; RH, relative humidity.

Since we found *TET* expression to be dynamic in the midbrain during EHC, we measured the concentration of its cofactor α-ketoglutarate (Figure 3A), which has been found to metabolically and epigenetically regulate macrophage inflammatory activation (Liu et al., 2017). The EHC group presented increased levels of α-ketoglutarate during conditioning (ED 10: t_6_ = 3.12, *P* = 0.02; ED 14: *t*_14_ = 3.76, *P* = 0.002), but not after the conditioning period (ED 18: *t*_12_ = 0.76, *P* = 0.46; Figure 3B). Next, we inhibited α-ketoglutarate accumulation during EHC by intra-ovo injection of BPTES, a glutaminase inhibitor, or saline into the blood vessels of the chorioallantoic membrane of ED 12 embryos. The embryos were returned to the incubator immediately after injection and continued their development until ED 14 and ED 18 (Figure 3C). To check the effectiveness of the inhibition, we first measured the concentration of α-ketoglutarate during conditioning in the embryo midbrains on ED 14, and after conditioning on ED 18 (Figure 3D). BPTES reduced the amount of midbrain α-ketoglutarate on both ED 14 (*t*_14_ = 5.05, *P* = 0.0002) and ED 18 (*t*_14_ = 2.69, *P* = 0.02; Figure 3D). TET family enzyme activity was also measured on ED 14 in saline- and BPTES-injected, non-conditioned and EHC chicks (Figure 3E). Multiple comparisons demonstrated a significant increase in TET activity in the saline-injected EHC group compared to the saline-injected controls (*P* < 0.0001), whereas there was no difference between BPTES-injected EHC and control groups (*P* = 0.99). There was also a strong interaction (*F*_2_, _23_ = 7.796, *P* = 0.003) between the BPTES effect (*F*_2_, _23_ = 29.34, *P* < 0.0001) and the conditioning effect (*F*_1_, _23_ = 21.45, *P* = 0.0001; Figure 3E). These results led us to conclude that α-ketoglutarate is necessary for TET enzyme activity, which is increased during EHC.

### TET Inhibition During EHC Has a Long-Lasting Effect, Blocking Heat Resilience Later in Life

**FIGURE 4:**
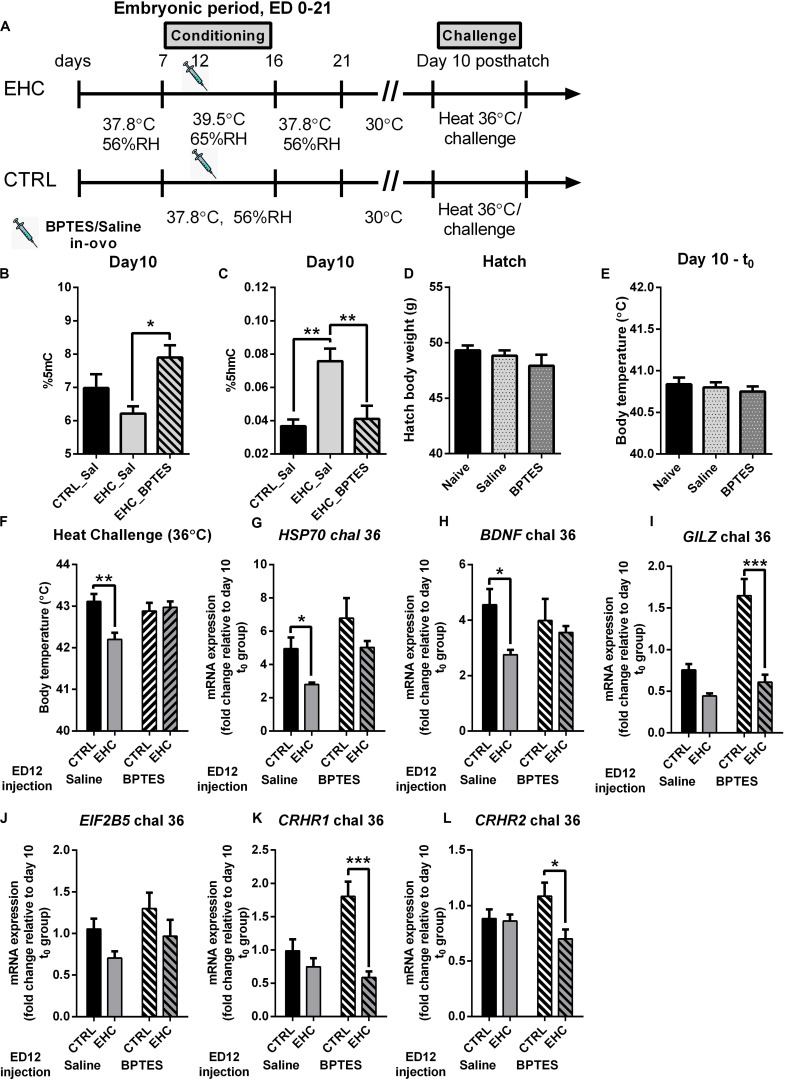
TET inhibition during EHC has a long-lasting effect, blocking heat resilience later in life. **(A)** Experimental scheme; 12.5 mg/kg BPTES or saline vehicle was injected on embryonic day (ED) 12, heat challenge was applied on day 10 post-hatch. **(B)** Total DNA%5mC in the hypothalamus on day 10 post-hatch, no challenge. EHC groups [n_*BPTES*_ = 6, n_*saline*_ (Sal) = 5, *P = 0.02]; control (CTRL)–Sal group (*n* = 7; vs. EHC–Sal group *P* = 0.24). **(C)** Total DNA%5hmC in the hypothalamus on day 10 post-hatch, no challenge. EHC groups [n_*BPTES*_ = 6, n_*saline*_ (Sal) = 7, **P = 0.006]; CTRL–Sal group (*n* = 7; vs. EHC–Sal group **P = 0.002). **(D)** Hatch body weight (g). Naïve (non-injected, *n* = 75), saline-injected group (*n* = 59) and BPTES-injected group (*n* = 51; *F*_2_, _182_ = 1.216, *P* = 0.299). **(E)** Day 10 t_0_ (before heat challenge) body temperature. Naïve (*n* = 8), saline-injected group (*n* = 17) and BPTES-injected group (*n* = 12; *F*_2_, _34_ = 0.365, *P* = 0.697). **(F)** Day 10 heat challenge (36°C). ED 12 saline-injected groups (n_*CTRL*_ = 8, n_*EHC*_ = 7, **P = 0.004); ED 12 BPTES-injected groups (n_*CTRL*_ = 6, n_*EHC*_ = 7, *P* = 0.99); EHC–BPTES group vs. EHC–saline group (*P = 0.02). **(G)** Hypothalamic mRNA expression of *HSP70* during day 10 heat challenge, normalized to *HMBS* and reported relative to their respective t_0_ controls. Groups injected with saline on ED 12 (n_*EHC*_ = 6, n_*CTRL*_ = 6, *P = 0.048); groups injected with BPTES on ED 12 (n_*EHC*_ = 6, n_*CTRL*_ = 6, *P* = 0.099). **(H)** Hypothalamic mRNA expression of *BDNF* during day 10 heat challenge, normalized to *HMBS* and reported relative to their respective t_0_ controls. Groups injected with saline on ED 12 (n_*EHC*_ = 6, n_*CTRL*_ = 6, *P = 0.039); groups injected with BPTES on ED 12 (n_*EHC*_ = 6, n_*CTRL*_ = 6, *P* = 0.80). **(I)** Hypothalamic mRNA expression of *GILZ* during day 10 heat challenge, normalized to *HMBS* and reported relative to their respective t_0_ controls. Groups injected with saline on ED 12 (n_*EHC*_ = 6, n_*CTRL*_ = 6, *P* = 0.137); groups injected with BPTES on ED 12 (n_*EHC*_ = 6, n_*CTRL*_ = 6, ***P < 0.001). **(J)** Hypothalamic mRNA expression of *EIF2B5* during day 10 heat challenge, normalized to *HMBS* and reported relative to their respective t_0_ controls. Groups injected with saline on ED 12 (n_*EHC*_ = 6, n_*CTRL*_ = 6, *P* = 0.245); groups injected with BPTES on ED 12 (n_*EHC*_ = 6, n_*CTRL*_ = 6, *P* = 0.277). **(K)** Hypothalamic mRNA expression of *CRHR1* during day 10 heat challenge, normalized to *HMBS* and reported relative to their respective t_0_ controls. Groups injected with saline on ED 12 (n_*EHC*_ = 6, n_*CTRL*_ = 6, *P* = 0.523); groups injected with BPTES on ED 12 (n_*EHC*_ = 6, n_*CTRL*_ = 6, ***P < 0.001). **(L)** Hypothalamic mRNA expression of *CRHR2* during day 10 heat challenge, normalized to *HMBS* and reported relative to their respective t_0_ controls. Groups injected with saline on ED 12 (n_*EHC*_ = 6, n_*CTRL*_ = 6, *P* = 0.980); groups injected with BPTES on ED 12 (n_*EHC*_ = 6, n_*CTRL*_ = 6, *P = 0.012). Data are presented as mean ± SEM. Significant effects between groups are indicated by *0.05 < *P* < 0.01, **0.01 < *P* < 0.001 using ANOVA test with Sidak’s multiple comparisons test. RH, relative humidity.

After establishing that TET is inhibited in the embryo by BPTES injection, we aimed to assess the long-term effect of embryonic TET inhibition (Figure 4A). Since the substrate for the TET family enzymes is 5mC, and a major part of its product is 5hmC, we measured the total%5mC and%5hmC on day 10 post-hatch, without heat/LPS challenge (Figures 4B,C). The EHC–BPTES group presented increased%5mC compared to the EHC–saline group (*P* = 0.02), but there was no difference in%5mC between the saline-injected control and EHC groups (*P* = 0.42; Figure 4B), indicating that BPTES impaired TET activity. The total 5hmC% was increased in the saline-injected EHC group compared to the saline-injected control group (*P* = 0.002). This increase in%5hmC was blocked by BPTES, as the BPTES-injected EHC group did not differ from the saline-injected control group (*P* = 0.96), and%5hmC was significantly lower than in the saline-injected EHC group (*P* = 0.006; Figure 4C). These results indicated that EHC activation of TET has a long-term effect, past the embryonic period.

Our next aim was to assess whether these epigenetic changes in TET activity during EHC underlie the heat resilience later in life. However, since BPTES inhibits glutaminolysis, and glutamate receptors in the preoptic hypothalamus play a role in thermal regulation (Sengupta et al., 2016), we wanted to determine whether BPTES affected other aspects of development. Therefore, we measured the chick’s body weight at hatch, and found no difference between naïve (non-injected), saline-injected or BPTES-injected chicks (*F*_2_, _182_ = 1.216, *P* = 0.299; Figure 4D). Next, we introduced 10-day-old chicks which had been previously injected with BPTES or saline during embryogenesis to heat challenge, and measured their body temperature before (t_0_; Figure 4E) and 6 h into the heat challenge (Figure 4F). BPTES did not have any effect on baseline body temperature (*F*_2_, _34_ = 0.365, *P* = 0.697; Figure 4E), further indicating that glutamatergic inhibition on ED 14 does not affect thermal regulation later in life. During heat challenge, there was a significant decrease in the saline-injected EHC group’s body temperature compared to the saline-injected control group (*P* = 0.004; Figure 4F), indicating thermal resilience, supported by a strong conditioning effect (*F*_1_, _24_ = 5.683, *P* = 0.03). The BPTES-injected EHC group presented higher body temperature than the saline-injected EHC group (*P* = 0.02), and no difference compared to the BPTES-injected control group (*P* = 0.99), indicating that BPTES blocked thermal resilience (Figure 4F). These findings are supported by the significant interaction (*F*_1_, _24_ = 8.371, *P* = 0.008) between BPTES and conditioning. It is worth noting that there was no difference between the BPTES-injected control and BPTES-injected EHC group body temperatures (*P* = 0.93) during the heat challenge, indicating that glutamatergic transmission affecting thermal regulation was not damaged by the *in-ovo* injection of BPTES in the embryonic period. In addition to body temperature, we measured mRNA expression, in the hypothalamus 6 h into the heat challenge, of heat and stress related genes including: *HSP70* which is an indicator of the chick’s heat sensitivity (Kisliouk et al., 2017); brain derived neurotrophic factor (*BDNF)* which is epigenetically regulated during heat conditioning (Katz and Meiri, 2006; Kisliouk and Meiri, 2009); glucocorticoid-induced leucine zipper (*GILZ*), involved in glucocorticoids anti-inflammatory effect (Riccardi, 2015); eukaryotic initiation factor 2b5 (*EIF2B5*) involved in the epigenetic regulation of thermal control establishment (Kisliouk et al., 2009); and corticotrophin release hormone receptors (*CRHR*) 1 and 2 which are increased in the hypothalamus of chicks during food deprivation (Kadhim et al., 2019). The expression of each group was normalized to the expression in a parallel t_0_ group which was not subjected to heat challenge. Multiple comparisons showed that increased expression of *HSP70* following heat challenge in the saline-injected EHC group was significantly lower (*P* = 0.048) than that in the saline-injected control group. However, *HSP70* expression in the BPTES-injected EHC group did not differ from that in the saline-injected controls (*P* = 0.94), and was significantly higher than that in the saline-injected EHC group (*P* = 0.042). There was a trend toward increased expression in the BPTES-injected EHC group compared to the BPTES-injected control group (*P* = 0.099), and a trend toward increased expression in the BPTES-injected control group compared to the saline-injected control group (*P* = 0.087). Although there was a strong BPTES effect (*F*_1_, _20_ = 7.902, *P* = 0.01), it did not interact (*F*_1_, _20_ = 0.072, *P* = 0.79) with the conditioning effect (*F*_1_, _20_ = 7.355, *P* = 0.01; Figure 4G). *BDNF* expression during heat stress was significantly lower in the saline-injected EHC group compared to the saline-injected control group (*P* = 0.039), however embryonic injection of BPTES attenuated this effect and there was no significant difference in *BDNF* expression between CTRL and EHC groups injected with BPTES (*P* = 0.8). Although there was a strong conditioning effect (*F*_1_, _20_ = 4.889, *P* = 0.04), there was no BPTES effect (*F*_1_, _20_ = 0.05, *P* = 0.82) nor interaction (*F*_1_, _20_ = 1.86, *P* = 0.19; Figure 4H). *GILZ* expression during heat stress was significantly lower in the BPTES-injected EHC group compared with the BPTES-injected CTRL group (*P* < 0.001), this reduction was not significant between the saline-injected control and EHC groups (*P* = 0.14). Nevertheless, there was a significant interaction (*F*_1_, _20_ = 9.68, *P* = 0.005), along with BPTES effect (*F*_1_, _20_ = 20.65, *P* < 0.001) as well as conditioning effect (*F*_1_, _20_ = 33.71, *P* < 0.001; Figure 4I). *EIF2B5* expression during heat stress did not differ between saline-injected CTRL and EHC groups (*P* = 0.24), nor between BPTES-injected CTRL and EHC groups (*P* = 0.28). Nevertheless, both saline and BPTES-injected EHC groups presented a tendency toward reduced expression compared to their controls, presenting a significant conditioning effect (*F*_1_, _20_ = 4.71, *P* = 0.04; Figure 4J). *CRHR1* expression during heat stress was significantly lower in the BPTES-injected EHC group compared with BPTES-injected CTRL group (*P* < 0.001), but there was no difference between saline-injected CTRL and EHC groups (*P* = 0.52). Overall, there was a strong interaction (*F*_1_, _20_ = 9.21, *P* = 0.007) along with significant conditioning effect (*F*_1_, _20_ = 20.34, *P* < 0.001) and a trend for BPTES effect (*F*_1_, _20_ = 4.15, P = 0.055; Figure 4K). *CRHR2* expression during heat stress was significantly lower in the BPTES-injected EHC group compared with BPTES-injected CTRL group (*P* = 0.012), but there was no difference between saline-injected CTRL and EHC groups (*P* = 0.98). There was a significant conditioning effect (*F*_1_, _20_ = 5.24, *P* = 0.033) along with a trend for interaction (*F*_1_, _20_ = 4.13, *P* = 0.056; Figure 4L).

### TET Inhibition During EHC Also Blocks Attenuation of the Inflammatory Response in the Hypothalamus, Later in Life

**FIGURE 5:**
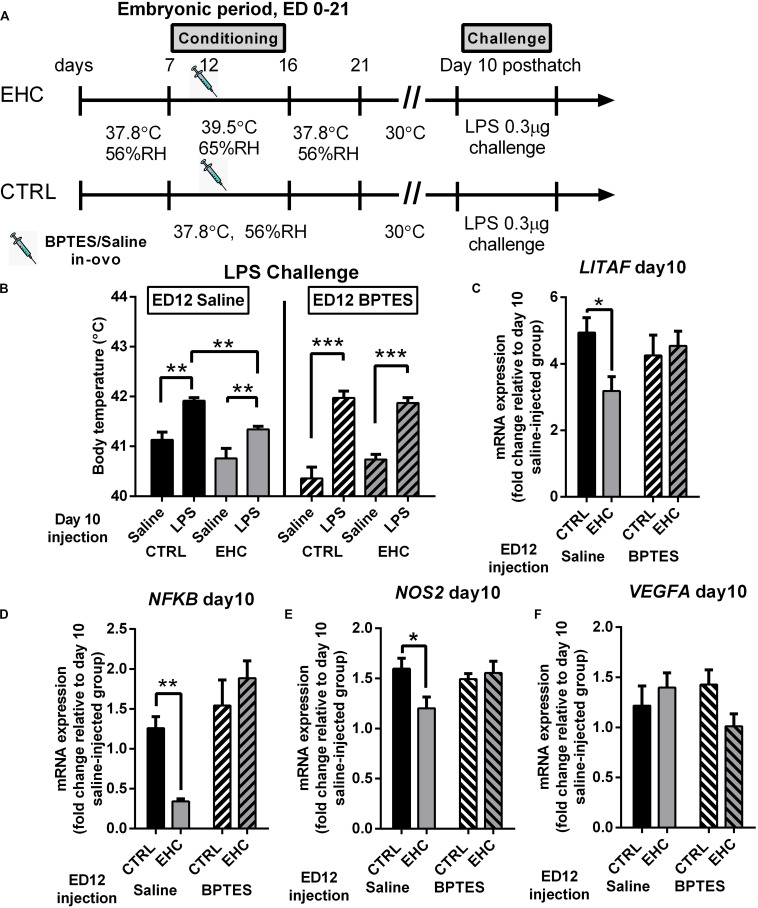
TET inhibition during EHC also blocks attenuation of inflammatory response in the hypothalamus, later in life. **(A)** Experimental scheme; 12.5 mg/kg BPTES or saline vehicle was injected on embryonic day (ED) 12, LPS challenge was applied on day 10 post-hatch. **(B)** LPS challenge on day 10 post-hatch. Groups injected with saline on ED 12 (left panel). Control (CTRL) groups (n_*LPS*_ = 6, n_*saline*_ = 7, **P = 0.003); EHC groups (n_*LPS*_ = 7, n_*saline*_ = 7, **P = 0.006); LPS–EHC group vs. LPS–CTRL group (**P = 0.007). Groups injected with BPTES on ED 12 (right panel). CTRL groups (n_*LPS*_ = 7, n_*saline*_ = 7, ***P < 0.0001); EHC groups (n_*LPS*_ = 7, n_*saline*_ = 5, ***P < 0.0001); EHC–LPS group vs. EHC–saline group (*P* = 0.62). **(C–F)** Hypothalamic mRNA expression was measured on day 10 post-hatch, 6 h after LPS or saline injection; fold change of *LITAF*, *NF*κ*B, NOS2*, and *VEGFA* was normalized to *HMBS* and reported relative to their saline controls. **(C)** *LITAF.* Groups injected with saline on ED 12 (n_*EHC*_ = 5, n_*CTRL*_ = 6, *P = 0.03); groups injected with BPTES on ED 12 (n_*EHC*_ = 6, n_*CTRL*_ = 6, *P* = 0.68). **(D)** *NF*κ*B.* Groups injected with saline on ED 12 (n_*EHC*_ = 6, n_*CTRL*_ = 6, **P = 0.005); groups injected with BPTES on ED 12 (n_*EHC*_ = 6, n_*CTRL*_ = 6, *P* = 0.25). **(E)** *NOS2.* Groups injected with saline on ED 12 (n_*EHC*_ = 6, n_*CTRL*_ = 6, *P = 0.024); groups injected with BPTES on ED 12 (n_*EHC*_ = 6, n_*CTRL*_ = 6, *P* = 0.895). **(F)** *VEGFA.* Groups injected with saline on ED 12 (n_*EHC*_ = 6, n_*CTRL*_ = 6, *P* = 0.667); groups injected with BPTES on ED 12 (n_*EHC*_ = 6, n_*CTRL*_ = 6, *P* = 0.144). Data are presented as mean ± SEM. Significant effects between groups are indicated by *0.05 < *P* < 0.01, **0.01 < *P* < 0.001, ***P < 0.0001 using ANOVA Sidak’s multiple comparisons test. RH, relative humidity.

Both the BPTES- and saline-injected groups were challenged with LPS on day 10 post-hatch (Figure 5A). The groups injected with BPTES (Figure 5B, right panel) presented a significant increase in body temperature in both the control (*P* < 0.0001) and EHC (*P* = 0.0006) groups 6 h into LPS challenge, but there was no difference in body temperature between the control- and EHC-BPTES groups during LPS challenge (*P* = 0.99). In addition, BPTES-injected groups presented only the LPS effect (*F*_1_, _22_ = 72.54, *P* < 0.0001), but no conditioning effect (*F*_1_, _22_ = 0.77, *P* = 0.39) or interaction (*F*_1_, _22_ = 2.24, *P* = 0.15). The groups that were injected with saline on ED 12 (Figure 5B, left panel), and challenged with LPS on day 10 post-hatch (19 days later) showed that although there was a significant increase in body temperature in both the EHC (*P* = 0.026) and control (*P* = 0.003) groups, the control group’s body temperature was significantly higher than that of the EHC group (*P* = 0.032), presenting a significant LPS effect (*F*_1_, _23_ = 26, *P* < 0.001), as well as a conditioning effect (*F*_1_, _23_ = 12.5, *P* = 0.002), with no interaction (*F*_1_, _23_ = 0.53, *P* = 0.47). These results indicated that while saline injection on ED 12 does not impair heat or inflammatory resilience, BPTES impairs both.

Next, we analyzed the influence of BPTES on proinflammatory (*LITAF*, *NF*κ*B*, *NOS2*, and *VEGFA*) and anti-inflammatory (*MRC1* and *IL10*) gene expression, 6 h into LPS challenge (Figures 5C–F). BPTES had no effect on *IL10* or *MRC1* expression (data not shown). The reduction in *LITAF* expression in the saline-injected EHC group compared to the saline-injected controls (*P* = 0.03) was not evident in the BPTES-injected groups (*P* = 0.68; Figure 5C). Similarly, the reduction in *NF*κ*B* expression in the EHC group compared to the saline-injected controls (*P* = 0.005) did not appear in the BPTES-injected groups (*P* = 0.25). Moreover, the BPTES-injected EHC group presented higher expression of *NF*κ*B* compared to the saline-injected EHC group (*P* < 0.0001; Figure 5D). *NOS2* expression, following LPS challenge, was reduced in the saline-injected EHC group compared to the saline-injected unconditioned controls (*P* = 0.02). However, there was no difference between the BPTES-injected control and EHC groups (*P* = 0.89), which displayed high expression similar to unconditioned saline injected controls. Two-way ANOVA revealed a significant interaction between the conditioning and BPTES effects (*F*_1_, _20_ = 5.067, *P* = 0.04; Figure 5E). *VEGFA* expression, did not differ between groups, and was not affected by conditioning or BPTES injection (Figure 5F). Overall, BPTES inhibition of TET activity interfered with the EHC-induced cross-tolerance to inflammation via upregulation of the expression of the proinflammatory genes *LITAF*, *NF*κ*B*, and *NOS2*.

### Locus Specific Hydroxymethylation and Methylation, in 10 Days-Old Chicks Is Dependent on Embryonic TET Activity

**FIGURE 6:**
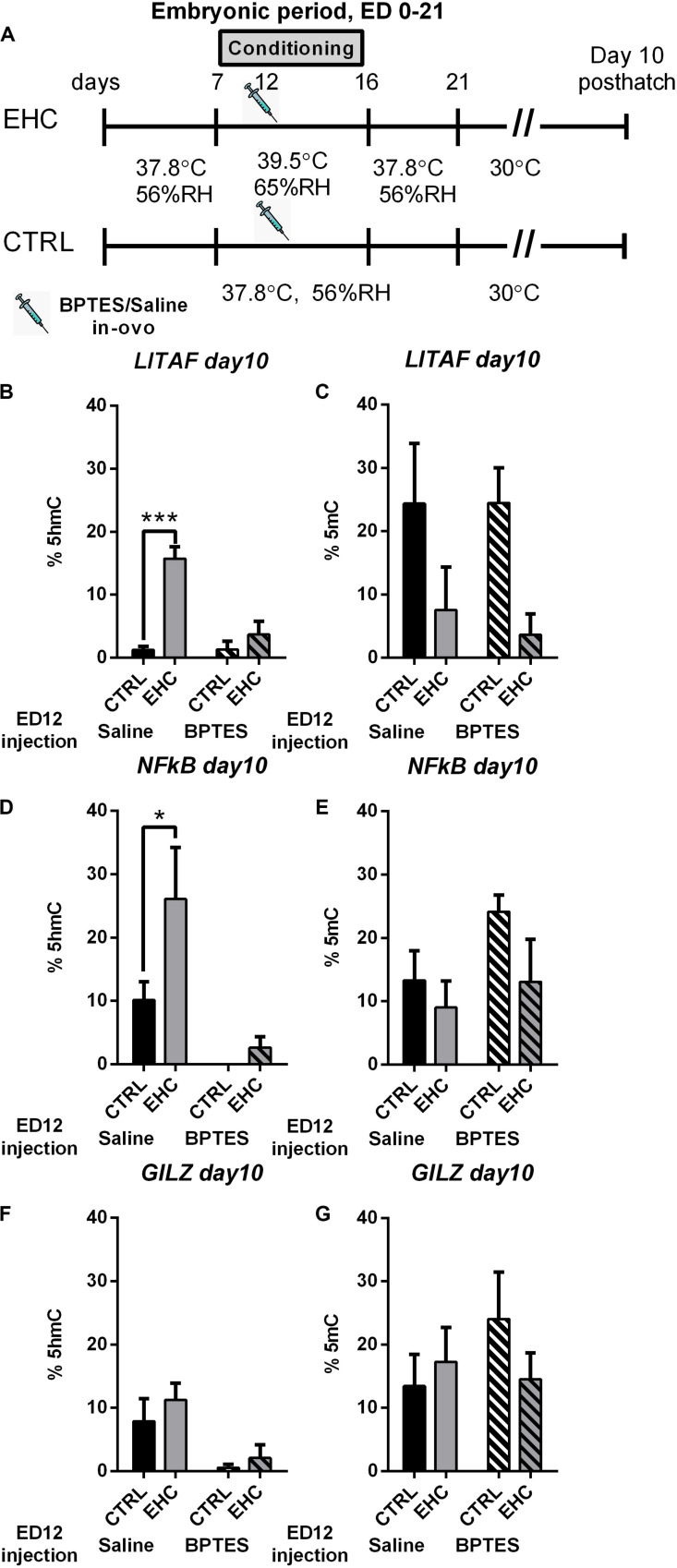
Locus specific hydroxymethylation and methylation, in 10 days-old chicks is dependent on embryonic TET activity. **(A)** Experimental scheme; 12.5 mg/kg BPTES or saline vehicle was injected on embryonic day (ED) 12, anterior hypothalamus was extracted on day 10 posthatch. **(B)** *LITAF* hydroxymethylation (%5hmC) on day 10 post hatch. Groups injected with saline on ED 12 (n_EHC_ = 6, n_CTRL_ = 6, ***P < 0.0001); groups injected with BPTES on ED 12 (n_EHC_ = 6, n_CTRL_ = 6, *P* = 0.52). **(C)** *LITAF* methylation (%5mC) on day 10 post hatch. Groups injected with saline on ED 12 (n_EHC_ = 6, n_CTRL_ = 6, *P* = 0.17); groups injected with BPTES on ED 12 (n_EHC_ = 6, n_CTRL_ = 6, *P* = 0.08). **(D)** *NFkB* hydroxymethylation (%5hmC) on day 10 post hatch. Groups injected with saline on ED 12 (n_EHC_ = 6, n_CTRL_ = 6, *P = 0.037); groups injected with BPTES on ED 12 (n_EHC_ = 6, n_CTRL_ = 6, *P* = 0.9). **(E)** *NFkB* methylation (%5mC) on day 10 post hatch. Groups injected with saline on ED 12 (n_EHC_ = 6, n_CTRL_ = 6, *P* = 0.78); groups injected with BPTES on ED 12 (n_EHC_ = 6, n_CTRL_ = 6, *P* = 0.22). **(F)** *GILZ* hydroxymethylation (%5hmC) on day 10 post hatch. Groups injected with saline on ED 12 (n_EHC_ = 6, n_CTRL_ = 6, *P* = 0.57); groups injected with BPTES on ED 12 (n_EHC_ = 6, n_CTRL_ = 6, *P* = 0.88). **(G)** *GILZ* methylation (%5mC) on day 10 post hatch. Groups injected with saline on ED 12 (n_EHC_ = 6, n_CTRL_ = 6, *P* = 0.17); groups injected with BPTES on ED 12 (n_EHC_ = 6, n_CTRL_ = 6, *P* = 0.08). Data are presented as mean ± SEM. Significant effects between groups are indicated by *0.01 < *P* < 0.05, **0.001 < *P* < 0.01, ***P < 0.001 using ANOVA Sidak’s multiple comparisons test. RH, relative humidity.

We found that embryonic inhibition of TET reduces total DNA hydroxymethylation, and influences the expression of proinflammatory genes as well as stress related genes in the hypothalamus. Therefore, we wanted to see if the changes in gene expression is supported by changes in locus specific DNA hydroxymethylation (%5hmC) and methylation (%5mC). To this end, we extracted DNA from the anterior hypothalamus of 10 day-old unchallenged cheeks that were either embryonically conditioned (EHC) or unconditioned and were injected with either BPTES or saline on ED 12 (Figure 6A), to study locus specific hydroxymethylation and methylation. Since we found TET dependent *LITAF*, *NFkB*, and *GILZ* changes in expression, during LPS or heat challenge, we chose these targets to assess locus specific hydroxymethylation and methylation. Genetic areas presenting possible transcriptional activity by the TFBIND algorithm, as well as enriched in CCGG motif, were chosen for analysis. *LITAF* CpG hydroxymethylation (%5hmC) and methylation (%5mC) was measured between NC_006101.5 461–645 (relative to transcription start), as this region contains 5 CCGG motifs as well as 126 TF binding sites with over 80% binding probability (Figures 6B,C). *LITAF* locus specific hydroxymethylation (%5hmC), was significantly increase in the saline-injected EHC group compared with saline injected controls (*P* < 0.0001), embryonic injection of BPTES, blocked hydroxymethylation, and both EHC and control groups presented reduced hydroxymethylation with no significant difference between groups (*P* = 0.52). Two-way ANOVA revealed a strong interaction (*F*_1_, _20_ = 14.48, *P* = 0.001), along with conditioning (*F*_1_, _20_ = 27.98, *P* < 0.0001) and BPTES (*F*_1_, _20_ = 14.22, *P* = 0.001) effects (Figure 6B). *LITAF* Locus specific methylation (%5mC) was not affected by BPTES, with significant conditioning effect (*F*_1_, _20_ = 7.96, *P* = 0.01; Figure 6C). *NFkB* CpG hydroxymethylation (%5hmC) and methylation (%5mC) was measured between NC_006093.5 78–200 (relative to transcription start), as this region contains 2 CCGG motifs as well as 75 TF binding sites with over 80% binding probability (Figures 6D,E). *NFkB* locus specific hydroxymethylation (%5hmC), was significantly increased in the saline-injected EHC group compared with saline-injected controls (*P* = 0.037). Embryonic injection of BPTES had a long lasting effect reducing%5hmC in both BPTES-injected groups, with no difference between control and EHC groups (*P* = 0.9). Moreover, BPTES-injected control group displayed 0%5hmC, in all subjects. Two-way ANOVA presented a conditioning (*F*_1_, _20_ = 4.43, *P* = 0.048) as well as a strong BPTES effect (*F*_1_, _20_ = 14.61, *P* = 0.001; Figure 6D). *NFkB* locus specific methylation (%5mC), was not affected by conditioning nor BPTES (Figure 6E). *GILZ* locus specific hydroxymethylation (%5hmC) and methylation (%5mC) was measured between NC_006091.5 4045–4248 (relative to transcription start), as this region contains 4 CCGG motifs as well as 126 TF binding sites with over 80% binding probability (Figures 6F,G). GILZ%5hmC did not differ between control and EHC saline-injected groups, there was also no difference between control and EHC BPTES-injected groups. However, BPTES injected groups presented reduced%5hmC, than saline injected groups and the effect of BPTES was significant (*F*_1_, _20_ = 11.13, *P* = 0.003; Figure 6F). *GILZ* locus specific methylation (%5mC), was not affected by conditioning nor BPTES (Figure 6G). Overall, the general effect of BPTES embryonic injection reducing total hydroxymethylation is also evident in specific targets along the TLR4/MyD88 inflammatory signaling pathway.

## Discussion

Early life stress can affect an organism’s future response to stress (Taylor, 2010). For example, according to Barker’s hypothesis, poor embryonic development leads to lifelong adversities (Barker, 1990; Hales and Barker, 1992). In this work, we focused on manipulations of the embryonic environment leading to stress cross-tolerance later in life. Cross-tolerance is a beneficial side effect of resilience of a completely different nature to another conditioned stressor (Horowitz, 2017). Here we demonstrated that EHC has long-term effects on inflammatory resilience in the hypothalamus. Furthermore, we revealed that heat–inflammatory cross-tolerance depends on the epigenetic increase of *TET* transcription and activity that occurs during the embryonic thermal manipulation and persists throughout life.

Across different species, heat acclimation has been shown to protect against a wide range of stressors (Shein et al., 2008; Umschwief et al., 2010, Umschweif et al., 2014; Assayag et al., 2012; Horowitz, 2017; Pollak et al., 2017; Hossain et al., 2018). Various findings point to the hypothalamus as the site of convergence of thermal–inflammation-related networks (Tzschentke, 2007; Tona et al., 2008; Tzschentke and Halle, 2009; Hueston and Deak, 2014; Loyau et al., 2014, 2016; Chao et al., 2015; Klett et al., 2015; Nassar et al., 2015; Audet et al., 2016; Morrison, 2016).

To demonstrate the concept of heat-conditioning leading to cross-tolerance involving the inflammatory response, we performed an LPS challenge on day 10 post-hatch by direct ICV injection of LPS into the brain. This approach was used so that the LPS effect would be directed to the hypothalamus, rather than to peripheral inflammation. The TLR4/MyD88 pathway was chosen to prove inflammatory resilience in the hypothalamus, because it has been previously shown to be induced during hypothalamic inflammation (Ropelle et al., 2010; Macrez et al., 2011; Valdearcos et al., 2015).

Therefore, we analyzed the mRNA expression of *LITAF* and *NF*κ*B* as proinflammatory markers, following LPS injection. We found, 6 h into the LPS challenge, induced febrile response and overexpression of *LITAF* and *NF*κ*B*, in addition to NFκB activation and nuclear translocation in the hypothalamus, all of which occur naturally during ischemic and high-fat-diet-induced hypothalamic inflammation (Macrez et al., 2011; Valdearcos et al., 2015). However, all of these effects were significantly reduced in the EHC group, along with increased expression of the anti-inflammatory genes *IL10* and *MRC1*. In the CNS, LPS binds to the TLR4 receptor and this activation is associated with neurodegenerative diseases (Xiang et al., 2015) and major depressive disorder (MDD). Furthermore, acute TLR4 stimulation activates the HPA axis (Liu et al., 2014), as well as being activated by alcohol binging (Crews et al., 2015). In chicks, HPA axis activation can be induced by heat-stress (Cramer et al., 2015), and indeed we see that LPS administration induces an increase in the chick’s body temperature, but to a lesser extent in the EHC model.

In this work, we studied the expression of both *TETs* and *DNMTs* during and after conditioning. Since we found *TET2* expression to be significantly increased in the EHC group, compared with controls, during conditioning (on ED 14),we chose to focus here on the role of TET family enzymes during EHC on the establishment of inflammatory resilience later in life. In a previous study, regarding the involvement of TET2 in cardiovascular disease, TET2 knockouts presented increased macrophage inflammatory activation, accelerating atherosclerosis (Fuster et al., 2017). Moreover, both *TET* and *DNMT* expression presented elevated levels during conditioning, which plummeted back to baseline after the conditioning period, suggesting that embryonic heat conditioning occurs at a time susceptible toward epigenetic modifications. Interestingly, *DNMT3A*, previously found to regulates *TET2* and *TET3* expression (Gong et al., 2016), presented increased expression both in the EHC and unconditioned groups, however, *TET2* and *TET3* expression was still elevated in both groups, during conditioning. This observation may suggest that although DNMT’s expression is elevated during EHC, TETs expression is more prominent. Both TET family enzymes and DNMT’s are regulated by metabolic intermediates such as α-keto-glutarate, S-adenosylmethionine (SAM), and Acetyl-CoA (Baardman et al., 2015), therefore epigenetic modifications are sensitive to metabolic regulation. Furthermore, embryonic heat conditioning exhibits long lasting metabolic effects in the fully grown chicken (Piestun et al., 2008, 2009).

In addition to our observations, previous studies have demonstrated that early life thermal conditioning induces stress resilience via the activation of TET enzymes (Cramer et al., 2018, 2019). Indeed, we found that EHC causes increased transcription of *TET1* and *TET2*, during conditioning, compared to controls. *TET3* expression was increased during the conditioning period in both groups, indicating its possible role during this developmental period. Moreover, *TET2* expression was significantly lower in the EHC group compared to controls at the beginning of the conditioning period (ED 10), but its enhanced transcription led to significantly higher expression on ED 14. In a recent study, *TET* expression in the hypothalamus was shown to be reduced in mice after birth (Cisternas et al., 2020), indicating a possible embryonic role.

These dynamic changes in *TET* family enzyme expression during the conditioning period led us to further study their involvement in the creation of EHC-induced cross-tolerance to inflammation. Interestingly, TET family enzymes have been previously reported to regulate different inflammatory events: e.g., activating inflammatory genes during macrophage differentiation and monocyte activation in monogenic inflammatory syndromes (Vento-Tormo et al., 2017); resolving inflammation in human peripheral blood mononuclear cells and murine peritoneal macrophages (Zhang et al., 2015); inducing cytokine expression in human dental pulp cells (Wang et al., 2018); and exhibiting TET-dependent anti-inflammatory cytokine regulation in human PMA-differentiated THP-1 cells and human primary mononuclear cells (Hassan et al., 2018), and adaptive immunity in zebrafish (Yang et al., 2016). However, little is known about the TET family enzymes’ role in regulating CNS inflammation. Nevertheless, the TET family enzyme cofactor α-ketoglutarate has been shown to be necessary for macrophage anti-inflammatory activation (Liu et al., 2017), for extracellular vesicle release during neuroinflammation (Wu et al., 2018) and also histone lysine demethylation (Niu et al., 2015). Therefore, we decided to measure the concentration of α-ketoglutarate in the midbrains of chick embryos during conditioning, and found it to be higher in the EHC group than controls. We then used BPTES to inhibit α-ketoglutarate synthesis. Reduced concentration of α-ketoglutarate in the midbrain was evident 2 and 6 days after BPTES injection, indicating TET inhibition from the moment of injection (ED 12) through the rest of the conditioning period. To assert BPTES inhibition of not only α-ketoglutarate production, but also TET activity, we measured TET activity in the midbrain during conditioning (ED 14). Indeed, we found that TET activity was higher in the saline-injected EHC group than the saline-injected controls, but also that the BPTES-injected EHC group did not present this increase in activity, which was similar to that in the BPTES-injected controls. After establishing TET inhibition by BPTES during the embryonic period, we studied the long-term effect of TET inhibition on day 10 post-hatch.

Since TET family enzymes are responsible for active demethylation of methylated CpGs (5mC) (Shi et al., 2017), we measured the total%5mC on day 10 post-hatch (almost 3 weeks after BPTES injection). The long-term effect of BPTES was evidenced by the higher abundance of the TET substrate%5mC in BPTES-injected chicks. Moreover, the TET product (%5hmC) in the BPTES-injected EHC group was lower on day 10 post-hatch than in the saline-injected EHC chicks, and did not differ from that in the saline-injected controls. This indicated that TET activity during the embryonic period has a long-term effect, which is still evident later in life.

Glutaminolysis is a general process implicating various cellular signaling pathways, among them glycolysis, and its inhibition influences embryonic metabolism (Vernardis et al., 2017). Therefore, we measured the chicks’ body weight at hatch, and found no significant differences between BPTES-injected, saline-injected and naïve (non-injected) chicks. Furthermore, during heat acclimation, glutamate receptors in the hypothalamus are necessary for the development of ischemic cross-tolerance (Yacobi et al., 2014; Matsuzaki et al., 2017). To ascertain that our results were a direct outcome of TET inhibition, and not of impaired hypothalamic development, we also measured baseline body temperature, prior to heat challenge (t_0_) and found no significant difference between groups. To further study the role of TET during the establishment of thermal resilience, we measured the chicks’ body temperature 6 h into the heat challenge and found that BPTES blocks heat resilience. It is also worth noting that BPTES did not change the body temperature of the challenged controls, indicating that the absence of thermal resilience in the BPTES-injected EHC group was a result of TET inhibition, rather than impaired glutamatergic transmission. Moreover, *HSP70*, shown to be a heat-stress-related marker (Kisliouk et al., 2017), presented higher expression in the BPTES-injected EHC group than in their saline-injected counterparts. We also found that both embryonic TET inhibition and embryonic conditioning affected the expression of the stress related genes *GILZ*, *CRHR1* and *CRHR2*, during heat challenge. The expression of *BDNF* and *EIF2B5*, which were previously shown to be epigenetically regulated during postnatal thermal establishment (Katz and Meiri, 2006; Yossifoff et al., 2008; Kisliouk and Meiri, 2009; Kisliouk et al., 2009), were affected during heat challenge by the embryonic conditioning but not by TET inhibition.

Finally, considering the effect of embryonic TET inhibition on the cross-tolerance to inflammation, we measured the chicks’ body temperature during LPS challenge; we found that EHC chicks injected with BPTES do not develop inflammatory cross-tolerance, as they displayed increased body temperature, and did not present the reduced expression of the proinflammatory genes *LITAF, NFkB* and *NOS2* found in saline-injected EHC chicks. *VEGFA* was not affected by the conditioning nor by TET inhibition.

To further study the specific effect of embryonic TET inhibition, in relation to the changes we found in gene expression later in life, we performed locus specific DNA methylation and hydroxymethylation analysis. To asses long-term effects, the analysis was performed on day 10 post-hatch on potential regulatory areas enriched with CpG’s that presented increased probability to bind transcription factors. Indeed, embryonic inhibition of TET is not transient, and has a long term effect reducing hydroxymethylation later in life. It should be noted that, reduction in hydroxymethylation was accompanied by an increase in methylation of the same locus. Nevertheless, this effect was less significant probably, due to the activity of DNMT’s.

Therefore, we suggest that TET-dependent epigenetic mechanisms that take place during the embryonic conditioning period downregulate inflammatory expression later in life.

The effect of TET family enzymes on inflammation is quite variable and still under investigation. Whereas there are studies showing specific TET-dependent increases in expression of the inflammatory cytokine *IL6*, as well as demethylation-dependent inflammation (Vento-Tormo et al., 2017; Itoh et al., 2018), others report TET-dependent reduction of inflammation and inflammatory cytokine expression via demethylation or histone deacetylation (Zhang et al., 2015; Chen et al., 2017; Hassan et al., 2018). Nevertheless, our findings of TET-dependent heat and inflammatory cross-tolerance further support our hypothesis that epigenetic mechanisms in the hypothalamus act, upon EHC, to create tolerance to inflammation. It is also worth noting that the effects on TET products and substrates, as well as locus specific effects and resilience, were measured 19 days after glutaminase inhibition, indicating a long-term effect.

This TET-dependent inflammatory resilience is supported by previous findings of inhibition of DNA methylation impairing the LPS-induced inflammatory response in mice (Matt et al., 2018). Furthermore, heat resilience in chicks induced by thermal conditioning on day 3 post-hatch was attenuated by the use of PARP inhibitor, which also inhibits TET family enzymes and reduces DNA hydroxymethylation (Cramer et al., 2019). Moreover TET activity is associated with oxygen metabolism (Matt et al., 2018; Skiles et al., 2018), and treating rats with hyperbaric oxygen has been found to protect against hypothalamic ischemia and inflammation caused by heat stroke (Tai et al., 2010), perhaps via induction of TET activity.

The main goal of this manuscript was to provide evidence that TET activity during heat conditioning at the embryonic period, is both necessary and sufficient to induce resilience to both thermal and inflammatory phenotypes later in life. The repertoire of proteins which are affected is probably very wide. Here, only for the proof of concept and to strengthen the phenotypic effect, we demonstrated a change in the expression of stress and thermal establishment related genes: *HSP70*, *BDNF*, *GILZ*, *EIF2B5*, *CRHR1*, and *CRHR2* as well as inflammatory-related genes i.e., *LITAF*, *NFkB*, *NOS2*, and *VEGFA*. It is intriguing to know the effect of embryonic heat conditioning on the entire genomic landscape, and to further analyze those genes to answer questions such as which target is transiently demethylated and which gene target will stay demethylated throughout the life span of the animals. These very interesting questions should be addressed in future research.

To conclude, we show that epigenetic regulation by TET family enzymes is pivotal in the establishment of cross-tolerance, by demonstrating that embryonic heat stress induces cross-tolerance to hypothalamic inflammation later in life. Furthermore, TET activity proved to be essential for induction of the protective effect. This phenomenon of early life epigenetic changes should be considered for the prevention of disease later in life.

## Data Availability Statement

The datasets generated for this study are available on request to the corresponding author.

## Ethics Statement

The animal study was reviewed and approved by the ethics committee of the Volcani Institute.

## Author Contributions

TR and NM designed and performed the experiments, generated the figures, interpreted the results, and wrote the manuscript. NM, TK, and SD supervised the project. TK, TC, and DS participated in performing the experiments, provided intellectual expertise, and helped interpret the results. All authors contributed to the article and approved the submitted version.

## Conflict of Interest

The authors declare that the research was conducted in the absence of any commercial or financial relationships that could be construed as a potential conflict of interest.

## Footnotes

1 http://tfbind.hgc.jp/
